# Dietary greenhouse gas emissions of meat-eaters, fish-eaters, vegetarians and vegans in the UK

**DOI:** 10.1007/s10584-014-1169-1

**Published:** 2014-06-11

**Authors:** Peter Scarborough, Paul N. Appleby, Anja Mizdrak, Adam D. M. Briggs, Ruth C. Travis, Kathryn E. Bradbury, Timothy J. Key

**Affiliations:** 1 British Heart Foundation Centre on Population Approaches for Non-Communicable Disease Prevention, Nuffield Department of Population Health, University of Oxford, Old Road Campus, Headington, Oxford, OX3 7LF UK; 2 Cancer Epidemiology Unit, Nuffield Department of Population Health, University of Oxford, Old Road Campus, Roosevelt Drive, Oxford, OX3 7LF UK

## Abstract

The production of animal-based foods is associated with higher greenhouse gas (GHG) emissions than plant-based foods. The objective of this study was to estimate the difference in dietary GHG emissions between self-selected meat-eaters, fish-eaters, vegetarians and vegans in the UK. Subjects were participants in the EPIC-Oxford cohort study. The diets of 2,041 vegans, 15,751 vegetarians, 8,123 fish-eaters and 29,589 meat-eaters aged 20–79 were assessed using a validated food frequency questionnaire. Comparable GHG emissions parameters were developed for the underlying food codes using a dataset of GHG emissions for 94 food commodities in the UK, with a weighting for the global warming potential of each component gas. The average GHG emissions associated with a standard 2,000 kcal diet were estimated for all subjects. ANOVA was used to estimate average dietary GHG emissions by diet group adjusted for sex and age. The age-and-sex-adjusted mean (95 % confidence interval) GHG emissions in kilograms of carbon dioxide equivalents per day (kgCO_2_e/day) were 7.19 (7.16, 7.22) for high meat-eaters ( > = 100 g/d), 5.63 (5.61, 5.65) for medium meat-eaters (50-99 g/d), 4.67 (4.65, 4.70) for low meat-eaters ( < 50 g/d), 3.91 (3.88, 3.94) for fish-eaters, 3.81 (3.79, 3.83) for vegetarians and 2.89 (2.83, 2.94) for vegans. In conclusion, dietary GHG emissions in self-selected meat-eaters are approximately twice as high as those in vegans. It is likely that reductions in meat consumption would lead to reductions in dietary GHG emissions.

## Introduction

Production, transport, storage, cooking and wastage of food are substantial contributors to greenhouse gas (GHG) emissions (Committee on Climate Change 2010; Garnett 2008; Intergovernmental Panel on Climate Change 2007). These GHG emissions include carbon dioxide (from fossil fuels used to power farm machinery and to transport, store and cook foods), methane (from enteric fermentation in ruminant livestock) and nitrous oxide (released from tilled and fertilised soils). Both methane and nitrous oxide are many times more potent GHGs than carbon dioxide and the majority of GHG emissions related to food are produced at the agricultural stage (Audsley et al. 2009; Garnett 2011). When measured by *consumption* (that is, all GHG emissions related to products consumed in the UK, regardless of where they were produced) food is responsible for approximately one fifth of all GHG emissions attributable to the UK (Berners-Lee et al. 2012; Garnett 2008). There is considerable variation in the amount of GHG emissions related to different food groups, with animal-based products generally having much greater emissions than plant-based products per unit weight (Audsley et al. 2009; Carlsson-Kanyama and Gonzalez 2009; Committee on Climate Change 2010; Gonzalez et al. 2011; Steinfeld et al. 2006). This is largely because of the inefficiencies involved in growing cereal crops to be used as animal feed, and methane produced in the digestive system of ruminants (Steinfeld et al. 2006). Although new technologies and changes in farming practices provide some scope for reduction in GHG emissions, substantial reductions can only be achieved through changes in consumption patterns and reduction in food waste (Stehfest et al. 2009; Weidema et al. 2008). This paper is concerned with differences in dietary GHG emissions between different diet groups within the UK and contributes to the debate regarding what constitutes a ‘healthy, sustainable diet’. Specifically, we use comparable data on actual diets of vegans, vegetarians, fish-eaters and meat-eaters to estimate the difference in dietary GHG emissions attributable to these four diet groups. Previous estimates of dietary GHG emissions for self-selected dietary groups have not compared meat consumers with those who abstain from meat (Vieux et al. 2013; Masset et al. 2014). Other estimates of GHG emissions by diet groups have been produced using modelled estimates of reduced meat diets (Baroni et al. 2007; Berners-Lee et al. 2012; Macdiarmid et al. 2012; Saxe et al. 2013; Vieux et al. 2012; Wilson et al. 2013) and may not reflect true differences in consumption behaviour between dietary groups.

## Methods

### Subjects and study design

The analyses are based on data from participants in the EPIC-Oxford cohort, which consists of 65,000 participants generally aged 20 and over at recruitment between 1993 and 1999 (Davey et al. 2003). Participants were resident in the UK and recruited through collaborating general practitioners, by post via vegetarian and vegan societies, and by adverts in vegetarian and health food magazines. Participants were also asked to recruit friends and relatives (‘snowballing’). A validated semi-quantitative food-frequency questionnaire (FFQ) that estimates intake (frequency of consumption) of 130 different food items over the previous 12 months (Bingham et al. 1994; Bingham et al. 1995) was completed at recruitment by most participants. For the analyses in this paper, we excluded individuals aged less than 20 years or 80 years and over at recruitment (*n* = 532), those who did not complete the FFQ or whose estimated daily energy intake from the FFQ was outside realistic values (men < 3.3 MJ or > 16.7 MJ, women < 2.1 MJ or > 14.7 MJ) or for whom more than 20 % of relevant food frequencies were unreported (*n* = 9,304), and 71 participants with unknown diet group, leaving a final analysis dataset of 12,666 males and 42,838 females.

### Classification of diet groups

For these analyses, six dietary groups were identified: high meat-eaters ( > = 100 g/d), medium meat-eaters (50 to 99 g/d), low meat-eaters ( > 0 and < 50 g/d), fish-eaters, vegetarians and vegans. Initially, subjects were categorised into meat-eaters, fish-eaters, vegetarians and vegans according to responses to the following yes/no questions:

- Do you eat any meat (including bacon, ham, poultry, game, meat pies, sausages)?
- Do you eat any fish?
- Do you eat any eggs?
- Do you eat any dairy products (including milk, cheese, butter, yoghurt)?

### Calculation of dietary GHG emissions

Nutritional analyses of the 130 food-item FFQ were based on nutritional data for 289 food codes taken from UK food composition tables, primarily the McCance & Widdowson series (Roe et al. 2002). We estimated the GHG emissions associated with these 289 food codes, measured as kgCO_2_e (that is, kg of GHG weighted by global warming potential over a 100 year time frame, with carbon dioxide weighted as 1, methane weighted as 25 and nitrous oxide weighted as 298) per 100 g of food. The method used was adapted from a previous investigation of the health impact of applying a carbon tax to foods in the UK (Briggs et al. 2013). The source document for GHG parameters was Audsley et al. (2009), which estimates comparable GHG emissions for 94 food commodities consumed in the UK. These parameters incorporate the life cycle of food commodities from the earliest stages of production to the retail distribution centre. Different parameters are estimated for foods produced in the UK, the EU and outside the EU. We produced single UK estimates of GHG emissions for each food commodity, weighted by current import and export patterns using 2007 food balance sheet data from the FAO (Food and Agriculture Organization. FAOSTAT. July 2013). The weighted estimates for the 94 food commodities are presented in the appendix. The GHG emissions for the 289 food codes were then constructed from these 94 parameters using two techniques which we describe below as ‘adjusting for density’ and ‘estimating GHG emissions for recipes’.

#### Adjusting for density

For cheese, fruit juices, dried fruit and soya milk, the weight of food that is consumed is not equivalent to the weight of food that is produced. GHG emissions parameters for these food categories were not available, so we applied adjustment factors to their primary commodity to account for the change in weight between production and consumption. Where possible these adjustment factors were taken from published life cycle analyses, but when these were unavailable we used information from specific food products. The adjustment factors that were applied are shown in Table 1.

**Table 1:** Adjustment factors for cheese, fruit juices, dried fruit and soya milk

Food	Related GHG emissions commodity	kgCO2e per 100 g of commodity	Adjustment factor	kgCO2e per 100 g of food	Rationale
Cheese	Milk	0.184	10.1	1.858	10.1 L of milk required for 1 kg semi-hard cheese (Berlin 2002)
Orange juice	Oranges	0.006	3.2	0.019	3.22 kg of oranges required for 1 kg of juice (combining natural and concentrated) (Beccali et al. 2009)
Apple juice	Apples	0.072	1.4	0.099	Copella website states ‘3lbs of apples are packed into every litre of apple juice’ (Copella. Copella fruit juices. http://www.copellafruitjuices.co.uk/our-juices/juices Accessed July 2013)
Soya milk	Soy beans	0.201	0.1	0.025	Recipe website suggests 160 g soy beans combined with 1.5-2 L of water (Spiciefoodie.com. Homemade soy milk. http://www.spiciefoodie.com/2013/01/14/homemade-soy-milk-or-how-to-make-soy-milk/Accessed September 2013)
Raisins	Grapes	0.083	3.2	0.264	Comparison of water content in grapes and raisins in UK food composition tables (Roe et al. 2002)

kgCO2e, kilograms of carbon dioxide equivalents (carbon dioxide has weighting 1, methane has weighting 25, nitrous oxide has weighting 298)

#### Estimating GHG emissions for recipes

For the remaining food codes a recipe was sought which was used to split the weight of the food between food commodities. This split was then used to construct a GHG emissions estimate using the GHG parameters of the original commodities. The UK food composition table (Roe et al. 2002) which provides nutritional data for the FFQ was used as the first source for identifying recipes. If no recipe was found then a Google search was performed with the food code name as the search term and the top website found with a recipe for the food was used. Where ingredients were identified that were not comparable to a food commodity (e.g. flaky pastry), then a recipe was identified for the ingredient using the same method. For each food code the weights of the ingredients were added in the order of heaviest to lightest, and a 90 % threshold was used such that once a value greater than 90 % was achieved the remaining ingredients were disregarded. This threshold was applied to remove small ingredients with unknown GHG emissions from the calculations (e.g. ‘a pinch of nutmeg’). We did not account for the cooking process (either at the industrial stage or at home) for any of the food codes.

For seven food codes (drinking chocolate, chocolate digestive biscuits, semi-sweet biscuits, Coffeemate, Horlicks powder, mixed toffees, and soft drinks) the use of a recipe seemed implausible given that these products are largely produced on an industrial scale. Here recipes were estimated (using percentages in the ingredients list where available) by a single researcher and agreed by the research team. A complete spreadsheet showing recipes and calculations for all 289 food codes is available upon request.

### Standardisation of diets to 2,000 kcal

GHG emissions for each individual were standardised to a 2,000 kcal daily diet (the level used for guideline daily energy intake for adults in the UK) so that differences in estimated energy consumption between diet groups do not affect the results. This was done for three reasons. Firstly, the FFQ does not allow for differences in portion sizes of fruit, vegetables and cereals which may differ between dietary groups and could exaggerate differences in energy intake between groups. Secondly, to address under and over-reporting that is common to diet studies (Scagliusi et al. 2008). Thirdly, so that the standardised results can be used to estimate the change in GHG emissions that would result from changing between dietary groups without changing the energy intake of the diet, which may be more relevant when considering the potential impact of changing diets on GHG emissions.

### Statistical analysis

The arithmetic means and standard deviations of dietary GHG emissions for each diet group were calculated. An ANOVA was conducted to estimate GHG emissions by diet group adjusted for sex and age, categorised in 10 years age bands from 20–29 to 70–79. Statistical significance was set at the 5 % level and all analyses were conducted using the Stata statistical package (StataCorp 2011).

## Results

The analysis dataset included 2,041 vegans, 15,751 vegetarians, 8,123 fish-eaters and 29,589 meat-eaters. Baseline characteristics by meat consumption are shown in Table 2. Meat-eaters tended to be older than fish-eaters, vegetarians and vegans. Assuming an ordered categorisation (high meat → medium meat → low meat → fish-eaters → vegetarians → vegans), there were significant trends towards lower total fat, saturated fat and protein consumption and higher carbohydrate, total sugar, fibre and fruit and vegetables consumption as animal-based food consumption decreased.

**Table 2:** Descriptive statistics

	Meat-eaters	High meat consumers ^a^	Medium meat consumers ^a^	Low meat consumers ^a^	Fish-eaters	Vegetarians	Vegans	p for trend
N	29,589	8,286	11,971	9,332	8,123	15,751	2,041	
Mean (SD) age at recruitment	49.1 (12.8)	49.7 (12.3)	49.8 (12.6)	47.5 (13.3)	41.8 (12.9)	38.6 (12.7)	37.3 (13.1)	< 0.0001
% female	76.9	72.1	77.8	80.0	82.2	76.9	63.4	0.13
Mean (SD) energy intake (kcal/d)	1,972 (535)	2,214 (528)	1,933 (500)	1,809 (508)	1,889 (526)	1,872 (528)	1,747 (554)	< 0.0001
Mean (SD) total fat (% energy)	31.6 (5.9)	33.2 (5.4)	31.4 (5.7)	30.5 (6.2)	30.7 (6.3)	30.5 (6.5)	28.0 (7.3)	< 0.0001
Mean (SD) saturated fat (% energy)	11.5 (3.3)	12.4 (3.1)	11.5 (3.2)	10.9 (3.4)	10.6 (3.3)	10.6 (3.4)	6.5 (2.1)	< 0.0001
Mean (SD) protein (% energy)	17.0 (3.0)	18.5 (3.1)	17.1 (2.8)	15.6 (2.5)	14.7 (2.4)	13.6 (2.1)	13.3 (2.3)	< 0.0001
Mean (SD) carbohydrate (% energy)	48.0 (6.2)	45.0 (5.7)	48.1 (5.7)	50.5 (6.3)	51.0 (6.5)	52.5 (6.6)	55.6 (7.8)	< 0.0001
Mean (SD) total sugars (% energy)	24.3 (5.7)	22.5 (5.1)	24.4 (5.4)	25.8 (6.3)	25.1 (6.3)	25.4 (6.3)	24.7 (8.7)	< 0.0001
Mean (SD) dietary fibre (NSP; g/d)	18.7 (6.8)	18.6 (6.2)	18.3 (6.5)	19.4 (7.4)	21.3 (7.5)	21.6 (7.8)	26.1 (9.3)	< 0.0001
Mean (SD) fruit and vegetables (80 g portions/d)	6.4 (3.5)	6.1 (3.2)	6.3 (3.2)	6.9 (4.0)	7.3 (3.9)	7.1 (3.9)	8.7 (5.6)	< 0.0001

*SD*, Standard deviation

^a^High meat = ≥100 g/d meat consumption; Medium meat = 50–99 g/d; Low meat = <50 g/d. p values indicate significance of trend along ordered categorisation high meat → medium meat → low meat → fish-eaters → vegetarians → vegans

Table 3 shows differences in dietary GHG emissions by diet group. The highest dietary GHG emissions were found in high meat-eating men and the lowest dietary GHG emissions were found in vegan women. The mean observed values of dietary GHG emissions for meat-eaters (results reported for women and then men) was 46 % and 51 % higher than for fish-eaters, 50 % and 54 % higher than for vegetarians and 99 % and 102 % higher than for vegans. The results of the ANOVA analysis showed highly statistically significant differences (*p* < 0.0001) in dietary GHG emissions between the six diet groups after adjustment for age and sex, with progressively higher emissions for groups with greater intakes of animal-based products.

**Table 3:** Mean greenhouse gas emissions per 2,000 kcal by diet type and sex

	Men (observed values)			Women (observed values)			Adjusted for age and sex	
	N	Mean dietary GHG emissions (kgCO2e)	SD	N	Mean dietary GHG emissions (kgCO2e)	SD	Mean dietary GHG emissions (kgCO2e)	95 % CIs
All meat-eaters	6,380	5.93	2.01	22,759	5.71	1.75		
High meat-eaters ( ≥ 100 g/day)	2,310	7.26	2.11	5,976	7.17	1.94	7.19	(7.16, 7.22)
Medium meat-eaters (50–99 g/day)	2,654	5.66	1.60	9,317	5.62	1.38	5.63	(5.61, 5.65)
Low meat-eaters ( < 50 g/day)	1,866	4.67	1.35	7,466	4.67	1.05	4.67	(4.65, 4.70)
Fish-eaters	1,448	3.94	1.12	6,675	3.90	0.88	3.91	(3.88, 3.94)
Vegetarians	3,641	3.85	1.29	12,110	3.80	0.93	3.81	(3.79, 3.83)
Vegans	747	2.94	1.25	1,294	2.87	0.90	2.89	(2.83, 2.94)

*SD*, Standard deviation; *CIs*, Confidence intervals; *N*, Number of Participants

kgCO2e, kilograms of carbon dioxide equivalents

## Discussion

We have shown that dietary GHG emissions associated with self-selected diets in the UK are strongly associated with the amount of animal-based products in the diet. After adjustment for sex and age, an average 2,000 kcal high meat diet had 2.5 times as many GHG emissions than an average 2,000 kcal vegan diet. This is the first study to demonstrate these differences in real self-selected diets of meat eaters and those who abstain from meat. There were also significant trends towards lower saturated fat, higher fibre and higher fruit and vegetable intake (but a higher intake of sugars) as the quantity of animal-based products in the diet decreases. Previous analyses of the same cohort have demonstrated lower BMI (Davey et al. 2003) and fewer ischaemic heart disease events (Crowe et al. 2013) in diet groups with lower intakes of animal products. Improved cardiovascular outcomes for vegetarian diets have been demonstrated in meta-analyses of cohort studies conducted in western populations (Huang et al. 2012; Key et al. 1999). Although observational studies are susceptible to residual confounding, non-systematic reviews of RCTs have demonstrated beneficial effects of plant-based diets on lipids (Ferdowsian and Barnard 2009) and weight status (Berkow and Barnard 2006). This suggests that advice to reduce the amount of meat and animal-based products in the diet would be consistent with the definition of a ‘healthy, sustainable diet’, although reductions of meat at the population level may pose nutritional challenges for key nutrients including iron and zinc which should be monitored (Millward and Garnett 2010).

A recent representative survey in the UK using four day weighted food diaries, the National Diet and Nutrition Survey 2008/10 (Bates et al. 2012), found that the average amount of meat consumed in adults aged 19–64 (including non-consumers) was 110 g/day, which suggests that the majority of adults in the UK would be categorised as ‘high meat consumers’ in our analysis. Reducing the amount of animal-based products in the diet represents an achievable way for an individual to reduce their carbon footprint. Assuming that the average daily energy intake in the UK is 2,000 kcal, then moving from a high meat diet to a low meat diet would reduce an individual’s carbon footprint by 920kgCO_2_e every year, moving from a high meat diet to a vegetarian diet would reduce the carbon footprint by 1,230kgCO_2_e/year, and moving from a high meat diet to a vegan diet would reduce the carbon footprint by 1,560kgCO_2_e/year. For context, an individual travelling on an economy return flight from London to New York has an addition to their carbon footprint of 960kgCO_2_e (Carbon Footprint. Carbon footprint calculator. www.carbonfootprint.com/calculator.aspx Accessed July 2013). A family running a 10 year old small family car for 6,000miles has a carbon footprint of 2,440kgCO_2_e (Carbon Footprint. Carbon footprint calculator. www.carbonfootprint.com/calculator.aspx Accessed July 2013), roughly equivalent to the annual carbon saving of two high meat eating adults moving to a vegetarian diet.

Two previous studies have estimated the difference in dietary GHG emissions of self-selected dietary groups (Vieux et al. 2013; Masset et al. 2014). Both of these studies were based on the representative Individual and National Survey on Food consumption in France. Vieux et al. (2013) showed that those who consumed a healthy diet, defined by low energy density, high nutrient density and low consumption of saturated fat, sugar and sodium, had higher dietary GHG emissions than those who consumed an unhealthy diet. Consumption of ruminant meat and pork, poultry and eggs was similar in both healthy and unhealthy diet groups. Masset et al. (2014) showed that diets with lower than average GHG emissions tended to be less healthy, defined using a nutrient density index. These low GHG diets consisted of approximately 20 % less meat, fish and eggs than the average diet. They also identified a subset of the population who consumed a healthy and low GHG emissions diet which did not cost more than an average diet and which was characterised by a higher content of plant-based products. In contrast to the study reported here, the authors did not directly compare diet groups defined by levels of meat consumption. Other studies have estimated the difference in dietary GHG emissions between diet groups, but have used modelled reduced meat dietary scenarios for comparison. The modelling has been conducted using three methods: diets have been constructed from scratch with reference to nutritionists (Baroni et al. 2007); observed meat-eating diets have been modified with selected ‘replacement foods’ (Berners-Lee et al. 2012; Saxe et al. 2013;); or optimisation programmes have constructed a low-meat diet that meets several pre-determined sustainability and nutritional criteria (Macdiarmid et al. 2012; Vieux et al. 2012; Wilson et al. 2013). Most of these modelling studies suggested that reducing animal-based products would reduce dietary GHG emissions (Baroni et al. 2007; Berners-Lee et al. 2012; Macdiarmid et al. 2012; Saxe et al. 2013), but one scenario considered by Vieux et al. (2012) suggested that reduced meat diets may *increase* dietary GHG emissions. In this scenario, meat was isocalorically replaced in the diet by fruit and vegetables - since the GHG emissions per 100 kcal of food in their database were generally higher for fruit and vegetables than for meat products, this resulted in an increase in GHG emissions. Saxe et al. (2013) found that a vegetarian diet in Denmark would reduce dietary GHG emissions compared to the average Danish diet by 27 %, compared to our estimate of 35 % reduction between meat eaters and vegetarians. The difference may be due to the criterion in the Saxe et al. analysis that the vegetarian diet should contain equal protein levels as the average Danish diet – in the EPIC-Oxford sample protein levels are significantly lower in vegetarians than in meat-eaters. By comparing the actual diets of self-selected diet groups, our results are not limited by restrictive criteria that may not be representative of true dietary behaviour. However, our data are based on cross-sectional comparisons between dietary groups, and as such they do not tell us how people would replace meat in the diet (although, the diet groups do at least represent diets that are actually consumed in the population). This is an important limitation that should be addressed by future longitudinal studies with repeated dietary measures.

The GHG emissions data used in this paper were developed specifically for this analysis. Although the nutrient intakes estimated by the FFQ have been validated against food diaries and some biomarkers (Bingham et al. 1994; Bingham et al. 1995), the GHG emissions have not. The GHG estimates for food items used in this paper are subject to uncertainty that is not captured in the confidence intervals shown in this paper – for example, LCAs of animal-based foods have shown considerable variation in GHG estimates, due to differences in methods used and in forms of agricultural production (Nijdam et al. 2012; de Vries and de Boer 2010; Roy et al. 2009). Estimates of total food-related GHG emissions using measurements of food consumption (such as the FFQ) are underestimates because they do not take account of food wastage. Throughout the analyses presented here we have assumed that GHG emission related to food wastage is reasonably similar across all food groups, but this may not be the case. Estimates of food wastage in the UK suggest that wastage of fruit and vegetables is higher than for meat products (Quested et al. 2013), which could reduce the difference in GHG emissions between the dietary groups. Consumption estimates derived from FFQs are also prone to under-reporting (Becker and Welten 2001; Scagliusi et al. 2008). Similarly, we have not adjusted for differences in raw and cooked weight of foods in these analyses. In most instances, such an adjustment results in smaller cooked weights, as it incorporates discarding inedible material (e.g. bones, skin, cores etc.), which would inflate the size of the dietary GHG estimates reported here.

The diets observed in the EPIC-Oxford cohort may not represent current consumption patterns in the UK. Firstly, they are taken from the baseline data collection period which was conducted in the 1990s. Secondly, a large proportion of the meat-eaters in the EPIC-Oxford cohort consists of family or friends of vegetarians and vegans, who are likely to have diets which differ from those of the general population in the UK. This difference can be assessed by comparing the amount of meat consumed in meat-eaters recruited through the snowballing technique with those recruited directly by participating GPs. This shows that 24 % of meat-eaters recruited via the snowballing technique were high meat consumers, compared to 41 % of meat-eaters recruited directly. Restricting the data by removing participants directly recruited makes little difference to the results (results not shown). Thirdly, the cohort tended to consume a healthier diet than the current UK population. For example, average consumption of fruit and vegetables in the EPIC Oxford cohort is over 6 portions per day, even in the meat eaters. The most recent National Diet and Nutrition Survey (Bates et al. 2012) estimated that average consumption in the UK was 4.1 portions per day.

Further work is needed to establish a defined ‘healthy, sustainable diet’, which should include the recommendation to reduce the consumption of animal-based foods. This is already a recommendation put forward by the Health Council of the Netherlands. Guidelines for a healthy diet: the ecological perspective. Health Council of the Netherlands: The Hague and The (2011) Although this study has only considered the impact of food on GHG emissions, reducing animal-based food consumption has also been proposed as a mechanism for achieving global food security given future trends in yield and agricultural land use (Foley et al. 2011; Godfray et al. 2010; Ray et al. 2013) and could also play a role in reducing water stress and biodiversity loss (Steinfeld et al. 2006). Although the epidemiological evidence largely supports the health-promoting effects of a vegetarian diet, this evidence is primarily drawn from observational studies and residual confounding cannot be ruled out. Further research is required to determine the longitudinal health effects of reducing animal-based products within the diet, preferably in randomised trials. This work should consider micronutrient intakes in vulnerable groups (Macdiarmid 2013; Millward and Garnett 2010) as well as risk factors for cardiovascular disease and cancer.

The current trend in the UK is towards increased meat consumption. The percentage of vegetarians and vegans in UK adults has decreased from an estimated 5 % in 2000/01 to 2 % in 2008/10, although these estimates are based on relatively small samples (Bates et al. 2012; Henderson et al. 2002). Over the same period FAOSTAT estimates that total meat supply in the UK increased from 78.5 kg/person/year to 84.2 kg/person/year (Food and Agriculture Organization. FAOSTAT. http://faostat3.fao.org/home/index.html#HOME Accessed July 2013), including an increase in consumption of beef – in 1961 (when FAOSTAT records began) total meat supply was only 69.2 kg/person/year. It is necessary to find strategies and interventions that can turn this trajectory around and support a population that is increasingly unfamiliar with a low meat diet.

## Conclusion

Analysis of observed diets shows a positive relationship between dietary GHG emissions and the amount of animal-based products in a standard 2,000 kcal diet. This work demonstrates that reducing the intake of meat and other animal based products can make a valuable contribution to climate change mitigation. Other work has demonstrated other environmental and health benefits of a reduced meat diet. National governments that are considering an update of dietary recommendations in order to define a ‘healthy, sustainable diet’ must incorporate the recommendation to lower the consumption of animal-based products.

## Appendix

**Table 4:** Greenhouse gas emissions for the 94 food commodities, weighted for production in the UK, imports from the EU, and imports from outside the EU

Food category	UK GHG emissions (kgCO2e/kg)
Alcoholic Beverages	3.3
Animal Fats	40.1
Apples	0.7
Aquatic Animals, Others	0.0
Aquatic Plants	0.0
Aquatic Products, Other	0.0
Bananas	1.4
Barley	3.8
Beans	0.8
Beer	3.8
Beverages, Alcoholic	3.5
Beverages, Fermented	2.4
Bovine Meat	68.8
Butter, Ghee	1.8
Cephalopods	5.4
Cereals - Excluding Beer	1.8
Cereals, Other	1.8
Citrus, Other	0.7
Cocoa Beans	3.4
Coconut Oil	2.1
Coconuts - Incl Copra	2.1
Coffee	10.1
Cream	2.4
Crustaceans	5.4
Dates	1.1
Demersal Fish	5.4
Eggs	4.9
Fats, Animals, Raw	35.6
Fish, Body Oil	0.0
Fish, Liver Oil	0.0
Fish, Seafood	5.4
Freshwater Fish	5.4
Fruits - Excluding Wine	0.9
Fruits, Other	0.9
Grapefruit	0.8
Grapes	0.8
Groundnut Oil	0.9
Groundnuts (Shelled Eq)	1.4
Honey	1.0
Lemons, Limes	0.6
Maize	0.7
Maize Germ Oil	0.4
Marine Fish, Other	5.4
Meat	35.9
Meat, Other	35.7
Milk - Excluding Butter	1.8
Molluscs, Other	0.0
Mutton & Goat Meat	64.2
Oats	1.0
Offals	35.9
Oilcrops	1.8
Oilcrops Oil, Other	2.4
Oilcrops, Other	2.3
Olive Oil	4.5
Olives	4.5
Onions	0.5
Oranges, Mandarines	0.6
Palm Oil	3.3
Palmkernel Oil	0.0
Peas	1.2
Pelagic Fish	5.4
Pepper	2.5
Pigmeat	7.9
Pimento	3.2
Pineapples	1.9
Plantains	1.6
Potatoes	0.4
Poultry Meat	5.4
Pulses	3.3
Pulses, Other	3.5
Rape and Mustard Oil	2.9
Rape and Mustardseed	2.9
Rice (Milled Equivalent)	3.9
Rye	1.0
Sesameseed	4.2
Sesameseed Oil	4.2
Soyabean Oil	1.8
Soyabeans	2.0
Spices	1.6
Spices, Other	1.6
Starchy Roots	0.4
Stimulants	0.0
Sugar & Sweeteners	0.1
Sugar (Raw Equivalent)	0.1
Sunflowerseed Oil	3.3
Sweeteners, Other	0.0
Tea	1.9
Tomatoes	1.5
Treenuts	2.0
Vegetable Oils	3.2
Vegetables	1.6
Vegetables, Other	2.2
Wheat	1.0
Wine	1.0
